# Comprehensive Characterization of Immunological Profiles and Clinical Significance in Hepatocellular Carcinoma

**DOI:** 10.3389/fonc.2020.574778

**Published:** 2021-01-22

**Authors:** Ping Tao, Liang Hong, Wenqing Tang, Qun Lu, Yanrong Zhao, Si Zhang, Lijie Ma, Ruyi Xue

**Affiliations:** ^1^ Department of Laboratory Medicine, Shanghai TCM-Integrated Hospital, Shanghai University of Traditional Chinese Medicine, Shanghai, China; ^2^ Department of General Surgery, Shanghai Fifth People’s Hospital, Fudan University, Shanghai, China; ^3^ Department of Gastroenterology and Hepatology, Shanghai Institute of Liver Diseases, Zhongshan Hospital, Fudan University, Shanghai, China; ^4^ Department of Biochemistry and Molecular Biology, School of Basic Medical Sciences, Fudan University, Shanghai, China; ^5^ Department of General Surgery, Zhongshan Hospital (South), Shanghai Public Health Clinical Center, Fudan University, Shanghai, China

**Keywords:** hepatocellular carcinoma, tumor microenvironment, landscape, heterogeneity, prognosis

## Abstract

**Background:**

Therapies targeting immune molecules have rapidly been adopted and advanced the treatment of hepatocellular carcinoma (HCC). Nonetheless, no studies have reported a systematic analysis between immunological profiles and clinical significance in HCC.

**Methods:**

We comprehensively investigated immune patterns and systematically correlated 22 types of both adaptive and innate immune cells with genomic characteristics and clinical outcomes based on 370 HCC patients from The Cancer Genome Atlas (TCGA) database through a metagene approach (known as CIBERSORT). Based on the *Quantitative Pathology Imaging and Analysis System* coupled with integrated high-dimensional bioinformatics analysis, we further independently validated six immune subsets (CD4^+^ T cells, CD8^+^ T cells, CD20^+^ B cells, CD14^+^ monocytes, CD56^+^ NK cells, and CD68^+^ macrophages), and shortlisted three (CD4^+^ T cells, CD8^+^ T cells, and CD56^+^ NK cells) of which to investigate their association with clinical outcomes in two independent Zhongshan cohorts of HCC patients (n = 258 and n = 178). Patient prognosis was further evaluated by Kaplan-Meier analysis and univariate and multivariate regression analysis.

**Results:**

By using the CIBERSORT method, the immunome landscape of HCC was constructed based on integrated transcriptomics analysis and multiplexed sequential immunohistochemistry. Further, the patients were categorized into four immune subgroups featured with distinct clinical outcomes. Strikingly, significant inter-tumoral and intra-tumoral immune heterogeneity was further identified according to the in-depth interrogation of the immune landscape.

**Conclusion:**

This work represents a potential useful resource for the immunoscore establishment for prognostic prediction in HCC patients.

## Introduction

Hepatocellular carcinoma (HCC) ranks as the fourth leading cause of cancer-related death worldwide (1). Amongst all precision medicine, immunotherapy is quickly becoming a leading option for modern cancer treatment. However, the effectiveness of targeted therapy applied in HCC, such as sorafenib (2) and regorafenib (3), is limited. Moreover, immune therapy has also been unsuccessfully tested in HCC for decades (4). Strikingly, blocking the interaction between programmed death-1 (PD-1) and programmed death-ligand 1 (PD-L1) has presented a substantial survival benefit in an open-label, non-comparative, phase 1/2 dose escalation and expansion trial in advanced HCC patients (5). Recently, for patients with unresectable hepatocellular carcinoma, atezolizumab combined with bevacizumab presented with better overall and progression­free survival outcomes than those taking sorafenib in the IMbrave150, a global, multicenter, open­ label, phase 3 randomized trial (6). Despite the promising advances made in tumor-immunotherapy, the effectiveness remains largely limited, which may be due to the complex dynamic nature and immune heterogeneity. To date, to make educated decisions and design novel combination treatment strategies, the comprehensive landscape of cells infiltrating the tumor microenvironment (TME) needs urgent elucidation.

The recruitment, distribution, and localization of immune cells in the TME vary in tumor centers. An increasing body of literature suggests a crucial role for the TME in cancer progression and therapeutic responses (7). A strong correlation between a high density of T-cell infiltration and favorable clinical outcome has been shown in several solid tumors, such as melanoma (8), colo-rectal (9), and breast cancers (10). Similarly, the intra-tumoral balance of regulatory and cytotoxic T cells was uncovered as a promising independent predictor for prognosis in HCC (11). In addition, a serial of driver genomic alterations was revealed by deep-sequencing on more than 1000 HCC patients (12). Moreover, a patient-derived cell line-based model, pharmacologic data, and genomic comparisons of multiple lesions in HCC demonstrated that intratumor heterogeneity affected the sensitivity of different therapeutic agents and tumor progression (13). Heterogeneity of TME can be influenced by numerous factors, including chemokines, cytokines, the permeability of the vasculature, and tumor cells themselves (14). Although TME has been recently analyzed in pan-cancer or HCC-specific settings, few studies can provide a comprehensive characterization of immunological profiles and clinical significance for HCC.

To identify TME infiltration patterns in HCC, previous studies attempted to used immunohistochemistry (IHC), which contained numerous types of immune cells including T cells, B cells, macrophages, monocytes, and NK cells (15). However, these IHC-based investigations typically used one specific monoclonal antibody to identify a given immune cell type (16). In addition, more reliable identification of immune cells can be discriminated by flow cytometry (16). However, there is limited information about the flow cytometry gating strategy, making it challenging to further analyze. Furthermore, as an alternative, continuously accumulating transcription data can provide an ideal resource for large-scale analysis of the immune landscape, and integrated computational methods and platforms have been developed to delineate the micro-anatomical components in TME.

To improve early prognosis prediction and comprehensively investigate the immune infiltration patterns in HCC, we integrated public transcriptomics data from The Cancer Genome Atlas (TCGA) and The Cancer Immunome Atlas (TCIA) database, and employed the algorithm “Cell Type Identification By Estimating Relative Subsets Of RNA Transcripts (CIBERSORT)” to demonstrate the immune gene expression profiles in the current study. As an open algorithm, CIBERSORT allows for high sensitivity and specific differentiation of 22 different human immune cell phenotypes using a machine-learning approach called support vector regression (17). Furthermore, it has successfully been used for immunologic model construction in several solid tumors (18).

In the present study, we used CIBERSORT to quantify the proportions of immune cells in 370 HCC patients based on their immune gene profiling available from public databases, and systematically correlated the TME phenotypes with clinical outcomes and pathologic features in HCC. Furthermore, we optimized a platform to quantify the immune infiltration patterns and inter-tumoral and intra-tumoral immune heterogeneity through *Quantitative Pathology Imaging and Analysis System*, and further independently validated six reproducible immune subsets and three immune subtypes according to specific gene expression patterns, molecular and cellular characteristics, and clinical outcomes. This work should represent a useful resource for the immunoscore establishment of patient prognosis and treatment selection in HCC.

## Materials and Methods

### Patients and Datasets

Two independent cohorts comprising 436 HCC patients who underwent curative resection at Zhongshan Hospital of Fudan University in 2006-2009 (training cohort, n = 258; validation cohort, n = 178) were retrospectively analyzed. The inclusion and exclusion criteria of patients, postoperative surveillance, and treatment modalities have been described previously (19). 56 spatially separated samples obtained from 14 primary HCC patients treated with curative resection were selected for the immune heterogeneity research. Overall survival (OS) and time to recurrence (TTR) were defined as the interval from the date of surgery to death and tumor recurrence, respectively (19). Patients without recurrence or death were censored at the last follow-up. The study was approved by the Research Ethics Committee of Zhongshan Hospital, with written informed consent obtained from each patient (19). Detailed clinicopathologic characteristics are summarized in **Supplementary Table S1**.

We systematically searched for HCC gene expression datasets that were publicly available and reported full clinical annotations. Patients without survival information were removed from further evaluation. In total, we gathered eight cohorts of samples from patients with HCC for this study: GSE25097 (n = 557, 268 HCC tumor, 243 adjacent non-tumor, 40 cirrhotic, and six healthy liver samples), GSE14520 (n = 488, 247 HCC tumor, 239 adjacent non-tumors, two healthy liver samples), GSE36376 (n = 433, 240 tumors and 193 adjacent non-tumors), GSE45267 (n = 87, 48 HCC tumors and 39 adjacent non-tumors), GSE6764 (n = 75), GSE41804 (n = 40), GSE60502 (n = 36), and TCGA-LIHC (n = 370). The raw data from the microarray datasets generated by Affymetrix and Illumina were downloaded from the Gene-Expression Omnibus (GEO; https://www.ncbi.nlm.nih.gov/geo/). The raw data for the datasets from Affymetrix were processed using the RMA algorithm for background adjustment in the Affy software package (20). RMA was applied to perform background adjustment, quantile normalization, and final summarization of oligonucleotides per transcript using the median polish algorithm. The raw data from Illumina were processed by the lumi software package (21). Level four gene-expression data (FPKM normalized) of The Cancer Genome Atlas (TCGA) were downloaded from the UCSC Xena browser (GDC hub: https://gdc.xenahubs.net). Data were analyzed with the R (version 3.4.0) and R Bioconductor packages (22). For TCGA data-sets, RNA-sequencing data (FPKM values) were transformed into transcripts per kilobase million (TPM) values, which are more similar to those resulting from microarrays and more comparable between samples (23). The TCGA database was included for all further analyses.

### Collection of Genomic and Transcriptomics-Related Data

The corresponding clinical data from these public databases were retrieved and manually organized when available. Updated clinical data and sample information for TCGA-LIHC samples were obtained from the Genomic Data Commons (https://portal.gdc.cancer.gov/) using the R package TCGA biolinks. Overall survival information, somatic mutation data, the numbers of predicted neoepitopes based on tumor-specific HLA typing, and total mutations for each HCC patient were obtained from the web portal (https://gdc.xenahubs.net).

### Evaluation of TME Infiltration Patterns

To systematically quantify the proportions of immune cells in the HCC samples, we applied the CIBERSORT algorithm and the LM22 gene signature, which allows for high sensitivity and specific discrimination of 22 human immune phenotypes, including T cells, B cells, NK cells, macrophages, monocytes, DCs, and MDSC subsets (17). CIBERSORT is a deconvolution algorithm that uses a set of reference gene-expression values (a signature with 547 genes) considered a minimal representation for each cell type and, based on those values, infers cell type proportions in data from bulk tumor samples with mixed cell types using support vector regression (17). Gene-expression profiles were prepared using standard annotation files, and data were uploaded to the CIBERSORT web portal (http://cibersort.stanford.edu/), with the algorithm run using the LM22 signature and 1,000 permutations. The range of infiltrating immune cells was calculated based on the CIBERSORT score, which was available in The Cancer Immunome Atlas (TCIA, https://tcia.at/). Detailed information on CIBERSORT is summarized in **Supplementary Table S2**.

### Multiplexed Staining, Imaging, and Multispectral Analysis

Sections were dewaxed and then rehydrated through a series of xylene to alcohol to distilled water washes. Heat-induced antigen retrieval (pH 6.0, Enzo Life Sciences, Inc. USA) was then performed by microwave antigen retrieval for 10 minutes. Next, sections were cooled to room temperature for 1 hour and transferred to Tris-buffered saline. Endogenous peroxidase was blocked using 0.3% H_2_O_2_ for 30 minutes and washed in 0.03% Tris-Buffered Saline-Tween-20 (TBST, Amersco) three times at 5 minutes each with gentle agitation. This was followed by incubation with a protein-blocking solution (Enzo Life Sciences, Inc. USA) for another 30 minutes to reduce nonspecific antibody staining. Then sections were incubated with the first primary antibody in a humidified chamber at 4°C overnight. After the first incubation, corresponding secondary horseradish peroxidase-conjugated polymer was used for antibody conjugation (Vector Laboratories, CA), and staining was detected using 1:200 dilution of fluorescein tyramide signal amplification (TSA) (Perkin Elmer, Waltham, MA) for 10 minutes. TSA visualization was performed with the Opal seven-color IHC Kit (NEL797B001KT; PerkinElmer) containing fluorophores DAPI, Opal 520, Opal 540, Opal 570, Opal 620, Opal 650, Opal 690, and TSA Coumarin system (NEL703001KT; PerkinElmer). After serial rinsing, slides were again placed in specific retrieval buffers in a microwave to remove redundant antibodies before the next staining as specified by the manufacturer (Perkin Elmer, Waltham, MA). After sequenced staining based on the protocol of each panel, nuclei were incubated and visualized with diamidino-phenyl-indole (DAPI) (Sigma-Aldrich) for 5 minutes, and sections were subsequently cover-slipped with fluorescence mounting media (Vector Labs, Burlingame, CA) and allowed to dry overnight. We optimized singles to duplex staining and so on until eventually a 7 plex panel was developed. CD20 was detected with 1:100 dilutions of Opal™520; CD56, 1:200 dilutions of Opal™540; CD4, 1:200 dilutions of Opal™570; CD68, 1:200 dilutions of Opal™620; CD14, 1:200 dilutions of Opal™650; and CD8, 1:50 dilutions of Opal™690. Detailed information of reagents and antibodies is provided in **Supplementary Table S3**.

Slides were scanned using the PerkinElmer Vectra^®^ 3 (PerkinElmer). Multispectral images were unmixed using spectral libraries built from images of single stained tissues for each reagent using the inForm advanced image analysis software according to the prearranged protocols. First, slides were imaged at 4x magnification based on DAPI and an automated inForm^®^ software algorithm (v. 3.0) was used to identify tissue areas of the slides. The tissue areas were then imaged at 10x magnification for channels associated with DAPI (blue), FITC (green), Cy3 (yellow), Cy5 (red), and TexasRed (red) for the multiple markers-stained slides to create RGB images. These images were processed by the automated inForm software enrichment algorithm to identify possible 20x high-power fields (HPF) of view according to the highest FITC/Cy3/Cy5/TexasRed. For each region, Vectra^®^ 3 Intelligent Slide Analysis System (Perkin Elmer, Waltham, MA) captured the fluorescent spectra every 10 nm of the emission light spectrum and combined these captures to create a single stack image.

Individual stained spectrum and auto-fluorescence were utilized to establish a spectral library, which consisted of the emitting spectral peaks of selected fluorophores for multispectral analysis by inform v3.2 software (Perkin-Elmer, USA). All computer pre-selected images were reviewed by a board-certified pathologist for acceptability; images lacking tumor cells, containing mainly necrotic cells, or abnormal fluorescence signals not expected with antibody localization or biology were rejected. Defining a pattern recognition learning algorithm, each fluorescence-labeled biomarker of nucleated cells can be individually identified according to unique fluorescence intensity. Each specific fluorescent spectral was used to generate pixel intensities for every pixel within the segmented region of interest through inform v3.2 software. Then, each fluorophore was spectrally unmixed into individual channels and saved as a separate file for analysis. This was done for positive or negative evaluation of each targeted biomarker, as well as the fluorescent intensity cutoff value, which was strictly defined depending on the FI threshold. FI threshold was set up by manual inspection of several representative fields (up to 5) and confirmed by corresponding bright field images. For cell phenotypic identification, the method of circle selection training was used for prejudged cells, and finally, an objective and consistent morphological identification standard was established.

### Quantification of Immune Cells’ Spatial Distribution

Based on these pixel intensities, MATLAB was used to analyze the information of individual cells through dimension reduction (detailed MATLAB code was obtained from https://lvdmaaten.github.io/tsne/). In each spectrally unmixed and phenotypic image, relative spatial distribution and location information of each single cell was defined as a bivariate point pattern. The fluorescence spectra of each individual cell were obtained by inform v3.2 software analysis. Unsupervised learning is employed to verify that the approach can partition the tissue sections according to distributional heterogeneity.

### Statistical Analysis

For comparisons of two groups, statistical significance for normally distributed variables was estimated by unpaired Student *t* tests, and non-normally distributed variables were analyzed by Mann-Whitney U tests (also called the Wilcoxon rank-sum test). For comparisons of more than two groups, Kruskal-Wallis tests and one-way analysis of variance were used as nonparametric and parametric methods, respectively. Correlation coefficients were computed by spearman and distance correlation analyses. Two-sided Fisher exact tests were used to analyze contingency tables. The Kaplan-Meier method was used to generate survival curves for the subgroups in each dataset, and the log-rank (Mantel-Cox) test was used to determine the statistical significance of differences. The hazard ratios for univariate analyses were calculated using a univariate Cox proportional hazards regression model. A multivariate Cox regression model was used to determine independent prognostic factors using the survminer package. All heatmaps were generated by the function of pheatmap (https://github.com/raivokolde/pheatmap). All statistical analyses were conducted using R (https://www.r-project.org/) or SPSS software (version 17.0), and the *P* values were two-sided. *P* < 0.05 was considered statistically significant.

## Results

### Integrated Analysis of Immune Cell Subpopulations Defining the Immunome Landscape of HCC

**Figure 1:**
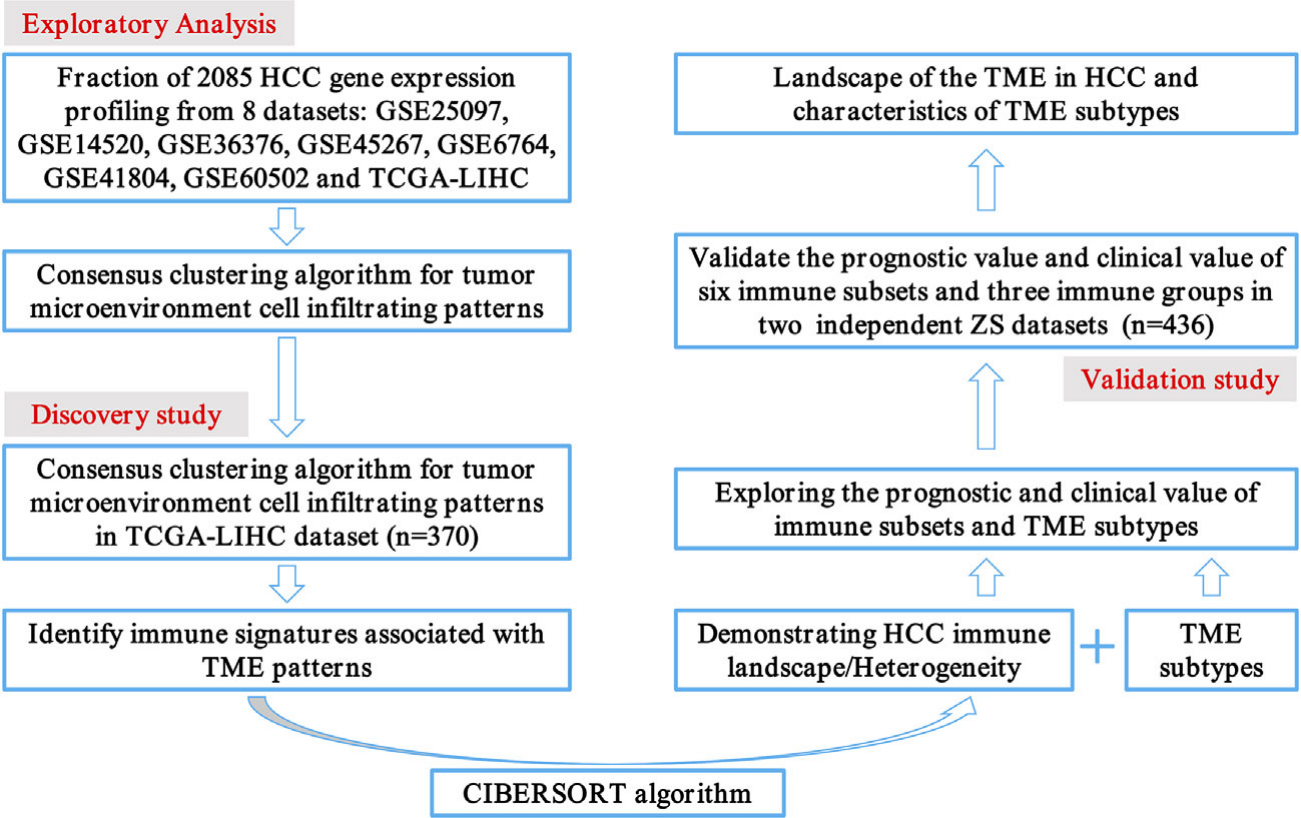
Flowchart presenting the overview of study design.

The transcriptome data of 2085 HCC patients were retrospectively selected from eight public datasets (GSE25097, GSE14520, GSE36376, GSE45267, GSE6764, GSE41804, GSE60502, and TCGA-LIHC). Detailed patient characteristics were obtained from the GEO (https://www.ncbi.nlm.nih.gov/geo/) and TCGA (https://portal.gdc.cancer.gov/) database. Immune cell-infiltrating patterns, signatures, patient selection scheme, and the workflow chart were systematically evaluated, as shown in **Figure 1**. To further characterize and understand the clinical differences among these immune subsets, we focused on the TCGA-LIHC cohort (n = 370), not only because it contained the paired tumor and non-tumors, but also because it provided the most comprehensive patient data. In addition, the CIBERSORT algorithm was more suitable to deconvolve microarray data from the Affymetrix platform (18).

**Figure 2:**
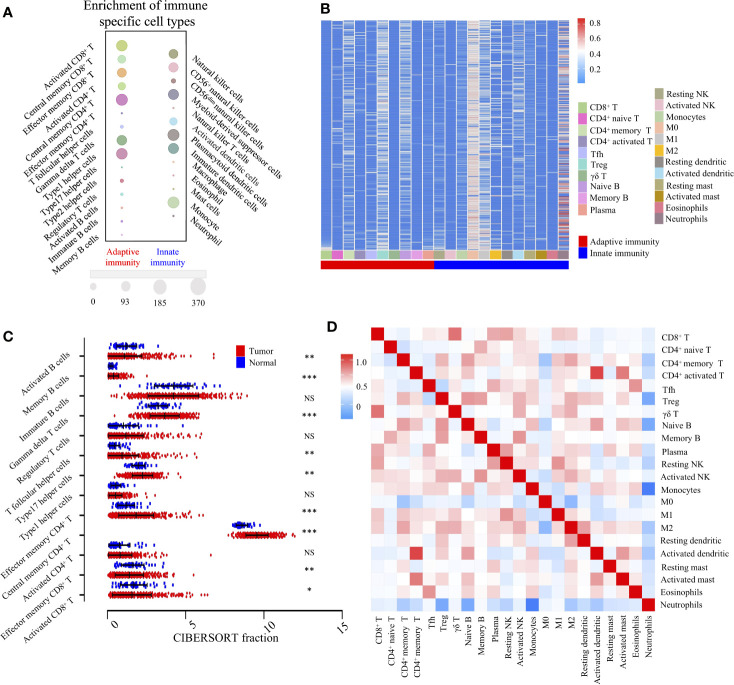
Landscape of TME and gene modules of the 22 immune subtypes in TCGA-LIHC cohort (n = 370). **(A)**. Bubble heatmap for comparison of two TME clusters, adaptive immunity, and innate immunity, comprising 28 immune cell fractions in TCGA-LIHC tumor tissues. The size of the circle represents the absolute value of the Z statistics. **(B)**. Unsupervised clustering of 22 TME cell types in the TCGA-LIHC cohort. TME clusters are shown as patient annotations. Columns represent TME cells and rows represent samples. Hierarchical clustering was performed with Euclidean distance and Ward linkage. **(C)**. Comparison of adaptive immune cells between tumor (red) and normal tissues (blue) in the TCGA-LIHC cohort, calculated using the CIBERSORT algorithm. *, **, *** denote *P* < 0.05, *P* < 0.01 and *P* < 0.001, respectively. NS denotes no significance (Mann-Whitney test). **(D)**. Correlation matrix followed by unsupervised hierarchical clustering in 22 immune subsets. Pearson correlation coefficients (R) were calculated. Correlation coefficients were plotted with negative correlation (blue), positive correlation (red), and R = 0 (white).

According to the transcriptomic information, cluster analysis was performed to reveal 28 distinct patterns of immune cell infiltration, as the TCGA-LIHC database presented. In adaptive immunity, the enrichment of central memory CD4^+^ T cells (370/370), Th1 cells (338/370), activated CD8^+^ T cells (338/370), γδ T cells (298/370), and effector memory CD8^+^ T cells (265/370) constituted the most abundant immune infiltrates in HCC, whereas the fraction of effector memory CD4^+^ T cells (1/370), Th17 cells (0/370), and tumor infiltrating-B cells (28/370) was significantly lower in HCC. Likewise, in innate immunity, plasmacytoid dendritic cells (370/370), monocytes (369/370), Myeloid-derived suppressor cells (MDSC) (320/370), and NK cells (268/370) were the four most common immune cell fractions, and the sum of their mean proportions was more than 60% in all clinical subgroups, whereas mast cells, eosinophils, neutrophils, and natural killer T cells indicated a sparse distribution, according to the Gene Set Enrichment Analysis (GSEA) (**Figure 2A**).

To investigate the cellular composition of the immune infiltrates in the TCGA-LIHC cohort, we further built the CIBERSORT inferred relative fractions of 22 immune cells. Interestingly, the percentage of macrophage (M2) (17.69%) was the highest, followed by resting memory CD4^+^ T cells (13.96%), γδ T cells (11.55%), and macrophages (M1) (7.29%) (**Figure 2B**). In adaptive immunity, student’s *t* test revealed that the percentages of activated B cells, monocyte, effector memory CD8^+^ T cells, and activated CD8^+^ T cells were decreased in intra-tumoral tissues, while the percentages of memory B cells, γδ T cells, Tfh cells, Th17 cells, effector memory CD4^+^ T cells, and central memory CD4^+^ T cells were increased (**Figure 2C**). In innate immunity, apart from macrophages, the percentages of neutrophils, eosinophils, mast cells, activated dendritic, immature dendritic cells, and monocytes were all decreased in intra-tumoral tissues. No significant differences were found in the comparison between normal and tumor tissues in activated NK cells and resting NK cells (**Supplementary Figure S1**). Furthermore, we investigated the coordination of immune cell fractions in the TCGA-LIHC cohort. Unsupervised hierarchical clustering of the immune cell correlation matrix was performed by visualized correlation analysis (**Figure 2D**). In summary, these results indicated a high degree of coordination of specific types of immune cells in HCC.

### Prognostic Landscape of Immunome Profile in HCC

**Figure 3:**
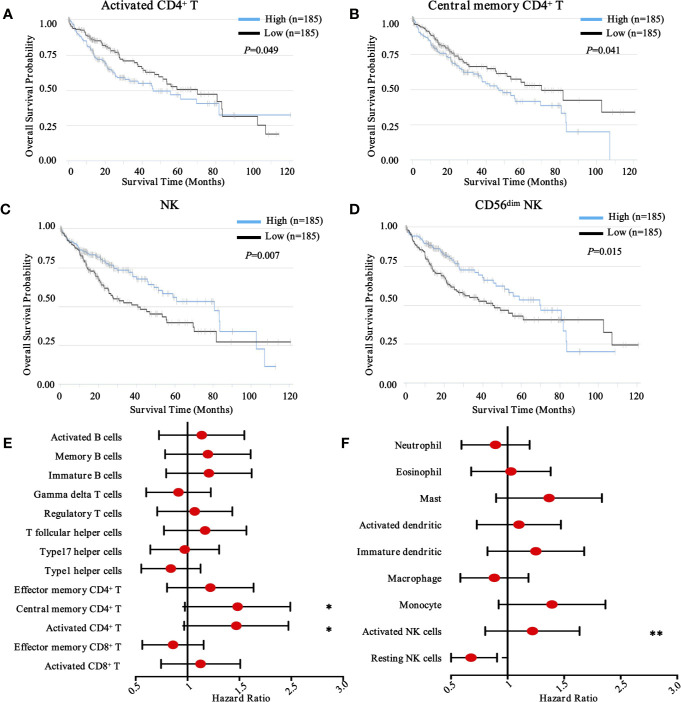
Prognostic landscape of immunome profile in TCGA-LIHC cohort (n = 370). **(A–D)**. Kaplan-Meier survival curve of tumor-infiltrating immune cells in LIHC (activated CD4^+^ T cell, central memory CD4^+^ T cell, NK cell, and CD56^dim^ NK cell). **(E, F)**. Forest plot showing the results of multivariate Cox regression analysis of 13 selected adaptive immunity cells and nine innate immunity cells in LIHC. *, ** denote *P* < 0.05 and *P* < 0.01, respectively.

To obtain a comprehensive prognostic evaluation of the immunome profile in HCC, we investigated the association between immune cells and prognosis through Kaplan-Meier analysis and log-rank test. Based on a mixed cell population using a gene expression-based approach from the TCIA database, we modified the RNA-Seq data, and cases were then divided into two groups based on the abundances of specific cell types. Notably, we found that more activated CD4^+^ T cell (HR = 1.41, 95% CI = 1.00 - 2.00, *P* = 0.049) and more central memory CD4^+^ T cell (HR = 1.42, 95% CI = 1.02 - 2.02, *P* = 0.041) subpopulations were associated with dismal prognosis in HCC (**Figures 3A, B**).

Conversely, in innate immunity, more activated NK cells (HR = 1.17, 95% CI = 0.83 - 1.66, *P* = 0.007) and CD56^dim^ NK cells (HR = 0.65, 95% CI = 0.46 - 0.92, *P* = 0.015) were associated with favorable prognosis in patients with HCC (**Figures 3C, D**). Meanwhile, other immune components, including T cell subsets, B cell subsets (**Figure 3E**), neutrophils, eosinophils, dendritic cells, macrophages, and monocytes, were also investigated (**Figure 3F**), although no significant differences were found. To further explore other immune prognostic signatures, a global prognostic map of other adaptive and innate immune components was summarized in **Supplementary Figure S2** and **S3**, respectively.

### Landscape of Immunomodulators and Their Prognostic Properties in HCC

Immune checkpoints consist of stimulatory and inhibitory molecules (24). Immune checkpoint blockade therapy has yielded promising clinical responses in patients with solid tumors (25), including HCC (26). To complement our immune population-centric survival analysis, we assembled an expression landscape of 22 immunomodulators and a global map of prognostic associations in HCC.

**Figure 4:**
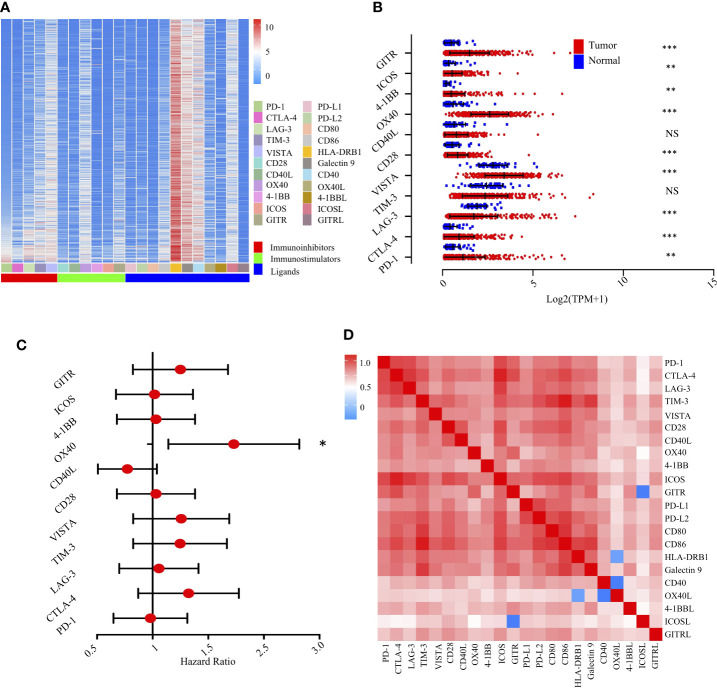
Landscape of the immune checkpoints and corresponding ligands in TCGA-LIHC cohort (n = 370). **(A)**. Unsupervised clustering of 22 immune checkpoints and specific ligands in tumor tissues. Columns represent receptors/ligands and rows represent samples. Hierarchical clustering was performed with Euclidean distance and Ward linkage. **(B)**. Comparison of immune checkpoints between tumor and normal tissues in TCGA-LIHC cohort. *, **, *** denote *P* < 0.05, < 0.01, and < 0.001, respectively. NS denotes no significance (Mann-Whitney test). **(C)**. Forest plot showing the results of multivariate Cox regression analysis of 11 selected immune checkpoints in LIHC. * denotes *P* < 0.05. **(D)**. Correlation matrix followed by unsupervised hierarchical clustering in 22 immune checkpoints and specific ligands in tumor tissues. Pearson correlation coefficients (R) were calculated. Correlation coefficients were plotted with negative correlation (blue), positive correlation (red), and R = 0 (white).

We first evaluated the expression of 22 immunomodulators, including co-inhibitory and co-stimulatory molecules (**Figure 4A**). Collectively, we found that PD-1, CTLA-4, VISTA, CD28, OX-40, 4-1BB, ICOS, and GITR were significantly increased in tumor tissues compared with non-tumor tissues. However, LAG-3 was significantly decreased (**Figure 4B**). Similarly, paired analysis of immune checkpoint ligands clearly demonstrated that ICOSL, 4-1BBL, OX40L, and Galectin 9 were significantly elevated in tumor tissues compared with non-tumor tissues. However, PD-L1 and PD-L2 were significantly decreased in tumor areas (**Supplementary Figure S4A**).

We then experimentally evaluated the reciprocal survival associations of immunomodulators in HCC. Using median as the cutoff value, we found that high OX40 (**Figure 4C**) and high OX40L (**Supplementary Figure S4B**) were significantly associated with poor prognosis, further indicating a potential clinical application for them as a prognostic tool (**Supplementary Figure S5**).

To extend the above findings, we investigated the coordination of immunomodulators in the TCGA-LIHC cohort. Unsupervised hierarchical clustering of immune cell correlation matrix was performed by visualized correlation analysis (**Figure 4D**). Notably, a reasonably high correlation among these immunomodulators was observed, further indicating a high degree of functional coordination of specific types of immunomodulators.

Immune checkpoint molecules were defined as essential “brake” molecules for transducing inhibitory signals into immune cells and regulating immune response and immune tolerance (27). Based on the TCGA-LIHC cohort, we performed correlation analysis between five immunoinhibitors and six distinct immune subpopulations. As shown in **Supplementary Figure S6A**, TIM-3 expression was significantly and positively correlated with the infiltration of dendritic cells (R = 0.68, *P* = 2.18e-47). Similarly, we further investigated the potential associations between six immunostimulators and immune subpopulations (**Supplementary Figure S6B**). Interestingly, ICOS was significantly and positively correlated with the infiltration of CD8^+^ T cells (R = 0.585, *P* = 8.37e-33). Correspondingly, cases exhibiting lower purity of tumor contents showed the elevated expression levels of all immune checkpoint genes. Conversely, cases rich in higher purities of tumor contents indicated reduced expression levels of these genes.

Taken together, tumor-associated molecules involved in immune checkpoints were often deregulated in TME, creating a way to escape immune surveillance. Our results indicated that heterogeneous molecular features were found in distinct immune subpopulations, thus providing unique therapeutic opportunities as depicted.

### Multiplexed Sequential Immunohistochemistry Enabling Defining Immune Landscape in HCC

**Figure 5:**
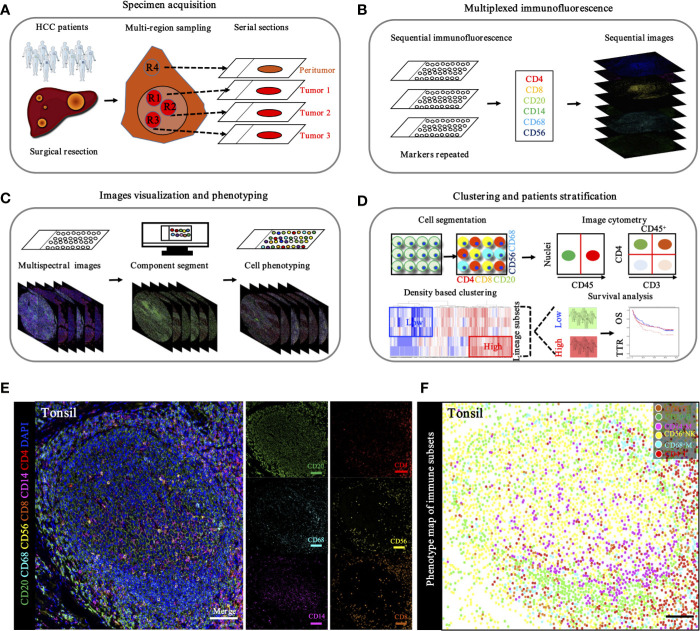
Schematic workflow of TME landscape characterizing through seven-plex IHC of key immuno-oncology phenotypic bio-markers. **(A)**. Specimen acquisition. Spatial multi-region sampling from FEEP sections. **(B)**. Multiplexed IHC staining with specific targets indicated distinct phenotypes: CD4, CD8, CD20, CD14, CD68, and CD56, which denoted CD4^+^ T cells, CD8^+^ T cells, CD20^+^ B cells, CD68^+^ macrophages, and CD56^+^ NK cells, respectively. **(C)**. Image visualization and phenotyping. Sequential steps, including single bio-markers, merged into composite image for enhanced target proteins visualization followed by inForm 2.1 (Perkin Elmer) based analysis including spectral unmixing, cell segmentation (nuclear/cytoplasmic/membrane) and cell phenotyping to precisely quantify immune evasion in tumor and adjacent peri-tumor. **(D)**. Clustering and patients’ stratification. Clustering and patients’ stratification were performed through obtained single-cell-based chromogenic signal intensity and computer based K-means algorithm learning partitioning. **(E)**. Immune landscape analysis of tonsil probed with a 7-plex panel labeling markers through multiplex IHC in tonsil. Scale bars, 100 μm. Representive images of individual channel in 7-plex panel, including CD4, CD8, CD20, CD14, CD68 and CD56, were presented. Scale bars, 100 μm. **(F)**. Phenotype panel demonstrates that seven fluorescent signals can be clearly unmixed *via* spectral imaging. Scale bars, 100 μm.

To *in situ* characterize the immune landscape through geographic distribution, a workflow based on the sequential IHC methodology was built, enabling simultaneous evaluation of multiple biomarkers in one FFPE section. After multi-region sampling from distinct HCC regions (**Figure 5A**), sequenced multiplex staining was performed on serial sections with standard preparation and incubation of primary and secondary antibodies (**Figure 5B**). Following this, slides were visualized by opal staining system and whole-slide digital scanning. Furthermore, serially scanning and digitized imaging were processed with a computational image analysis workflow after all rounds were finished (**Figure 5C**). Finally, in-deep analysis, like clustering and patients’ stratification, was performed based on the corresponding algorithms and projects (**Figure 5D**).

Based on the hypothesis that specific gene signatures could predict individual immune cell subsets, we first evaluated the ability of our single and sequenced multiplex staining assessment to differentiate immune cell subsets in tonsil tissues, as the positive control (**Figure 5E**). Consistent with this hypothesis, corresponding visualization and phenotyping were able to significantly differentiate subpopulations within complicated immune microenvironments (**Figure 5F**).

**Figure 6:**
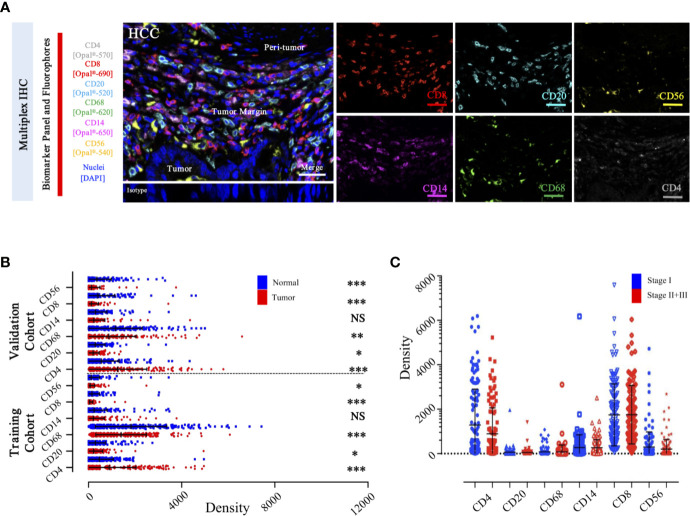
Integrated and multiplexed IHC detection of immune landscape in ZS-HCC cohorts. **(A)**. Seven-plex multispectral IHC images depict the immune landscape in HCC with tumor, peri-tumor, and margin invasion areas. (Iso) isotype-matched control antibody. Detailed single spectral image of seven-color panel, CD8 (red), CD68 (green), CD4 (white), CD56 (yellow), CD20 (cyan), CD14 (magenta), and DAPI (blue). Scale bars, 100 μm. **(B)**. Comparison and distribution of the six-immune features between tumor and paired normal tissues in the training cohort (n = 258) and validation cohort (n = 178). *, **, *** denote *P* < 0.05, *P* < 0.01, and *P* < 0.001, respectively. NS denotes no significance (Mann-Whitney test). **(C)**. Associations of the six-immune features in tumor areas with patients’ TNM stage in training cohort (n = 258).

To specifically describe the complexity and phenotype of the immune profile in HCC tumor centers, we established and validated the seven-color panel in paired tumor and non-tumor liver tissues from two independent Zhongshan cohorts comprising 436 HCC patients and encompassing six distinct epitopes to phenotype lymphoid lineage cells (**Figure 6A**). This panel depicted and visualized CD4^+^ T cells, CD8^+^ T cells, CD20^+^ B cells, CD14^+^ monocytes, CD56^+^ NK cells, and CD68^+^ macrophages, respectively. The level of immune features was defined as the median density of positively stained cells in two specific regions of each HCC patient. Consistent with the transcriptome data from the TCGA-LIHC cohort, a significantly higher density of CD8^+^ T cells (median, 46 vs 20 cells/mm^2^, *P* < 0.0001), CD20^+^ B cells (median, 145 vs 133 cells/mm^2^, *P* = 0.0475), CD56^+^ NK cells (median, 10 vs 6.5 cells/mm^2^, *P* = 0.0276), and CD68^+^ macrophages’ (median, 1738 vs 1358 cells/mm^2^, *P* < 0.0001) infiltration were found in the non-tumor versus tumor liver comparison in training cohort. Conversely, significantly higher infiltration of CD4^+^ T cells (median, 356 vs 308 cells/mm^2^, *P* < 0.0001) was noted in tumor liver tissues than non-tumor tissues. However, no significant difference was found in tumor versus non-tumor liver comparison of CD14^+^ monocytes (median, 30 vs 41.5 cells/mm^2^, *P* = 0.4067) (**Figure 6B**). Analogously, similar results were fully confirmed by the validation cohort (**Figure 6B**). In addition, we further investigated the frequency of distinct immune features in early and advanced stages in the training cohort, but no significant differences were found (**Figure 6C**). These findings demonstrated a dynamic change of local immune status in HCC. In particular, the significant difference in immune infiltration implied that pro-tumorigenic and anti-tumorigenic components are happening in TME.

### Prognostic Significance of Distinct immune Subgroups

**Figure 7:**
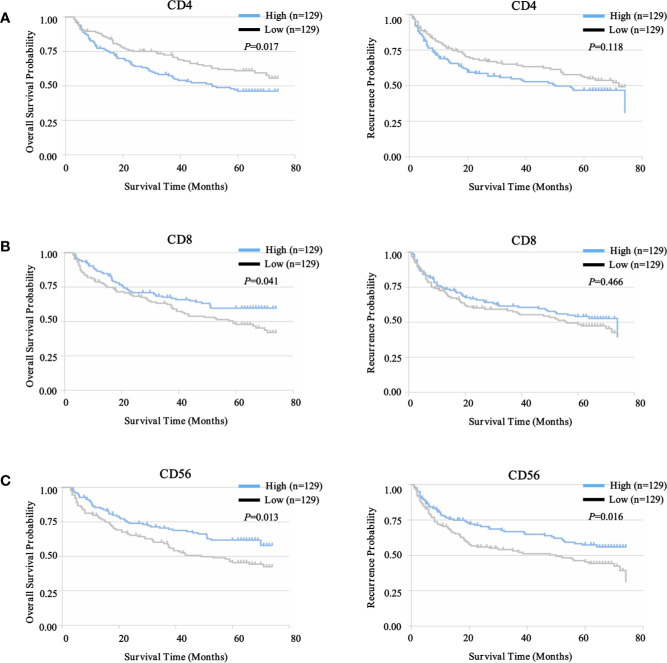
Prognostic landscape of immunome profile in ZS-HCC training cohort (n = 258). **(A)**. Kaplan-Meier analysis of OS and TTR for tumor-infiltrating CD4^+^ T cell subset based on cell density. **(B)**. Kaplan-Meier analysis of OS and TTR for tumor-infiltrating CD8^+^ T cell subset based on cell density. **(C)**. Kaplan-Meier analysis of OS and TTR for tumor-infiltrating CD56^+^ NK cell subset based on cell density.

To select prognostic immune features, we first sought to define whether the infiltration of six immune cell types was related to patient survival. We calculated the density of defined subpopulations for each patient and stratified them into high and low groups according to the median value. Strikingly, a higher density of CD4^+^ T cells was significantly associated with unfavorable OS (*P* = 0.017), but not TTR (*P* = 0.118) (**Figure 7A**). In contrast, a higher density of CD8^+^ T cells was significantly associated with favorable OS (*P* = 0.041), but not TTR (*P* = 0.466) (**Figure 7B**). Moreover, more infiltration of CD56^+^ NK cells harbored a significantly prolonged OS (*P* = 0.013) and TTR (*P* = 0.016) (**Figure 7C**). However, the high density of CD14^+^ monocytes, CD68^+^ macrophages, and CD20^+^ B cells neither correlated with patients’ OS and TTR (**Supplementary Figure S7**). Similar prognostic significance of these immune subgroups was also confirmed in the validation cohort (**Supplementary Figure S8**). Then, we conducted the univariate and multivariate analysis of prognostic factors associated with OS and TTR in the Zhongshan cohort (n = 258). Multivariate analysis revealed that the association of a high density of CD4^+^ T cells with unfavorable OS (HR = 1.480, 95% CI = 1.009 - 2.173, *P* = 0.045) was independent of AFP level, tumor size, tumor differentiation, microvascular invasion, and TNM stage (**Table 1**).

**Table 1:** Univariate and multivariate analysis of prognosis factors associated with OS and TTR in Zhongshan cohort (n=258).

Variables	OS				TTR			
****	Univariate	Multivariate			Univariate	Multivariate		
	*P*	HR	95%CI	*P*	*P*	HR	95%CI	*P*
Age, years (>51 vs. ≤51)	0.951			NA	0.727			NA
Gender (Male vs. Female)	0.492			NA	0.843			NA
HBsAg (Negative vs. Positive)	0.081			NA	0.305			NA
Liver cirrhosis (No vs. Yes)	0.564			NA	0.068			NA
Serum AFP, ng/ml (>20 vs. ≤20)	**0.001**	**1.564**	**1.039–2.353**	**0.032**	**0.012**	1.396	0.948–2.054	0.091
Serum ALT, U/L (>75 vs. ≤75)	0.462			NA	0.380			NA
Tumor size (cm) (>5 vs. ≤5)	**<0.001**	**2.237**	**1.429–3.504**	**<0.001**	**<0.001**	1.346	0.882–2.052	0.168
Tumor multiplicity (Multiple vs. Single)	0.231			NA	0.072			NA
Tumor differentiation (Poor vs. Well)	**<0.001**	**1.636**	**1.107–2.417**	**0.014**	**0.012**	1.205	0.810–1.791	0.357
Microvascular invasion (Yes vs. No)	**<0.001**	**2.015**	**1.322–3.072**	**0.001**	**<0.001**	**1.925**	**1.245–2.975**	**0.003**
TNM stage (II–III vs. I)	**<0.001**	**1.454**	**0.910–2.321**	**0.117**	**<0.001**	**1.633**	**1.014–2.631**	**0.044**
CD4^+^ T (High vs. Low)	**0.017**	**1.480**	**1.009–2.173**	**0.045**	0.118			NA
CD8^+^ T (High vs. Low)	**0.041**	1.035	0.654–1.637	0.883	0.466			NA
CD14^+^ Monocyte (High vs. Low)	0.245			NA	0.432			NA
CD20^+^ B (High vs. Low)	0.09			NA	0.329			NA
CD56^+^ NK (High vs. Low)	**0.013**	0.792	0.499–1.256	0.321	**0.016**	1.205	0.810–1.791	0.139
CD68^+^ Macrophage (High vs. Low)	0.211			NA	0.999			NA

OS, overall survival; AFP, alpha-fetoprotein; ALT, alanine aminotransferase; TNM, tumor-nodes-metastases; HR, hazard ratio; CI, conﬁdential interval; NA, not available. The bold indicates P < 0.05.

**Figure 8:**
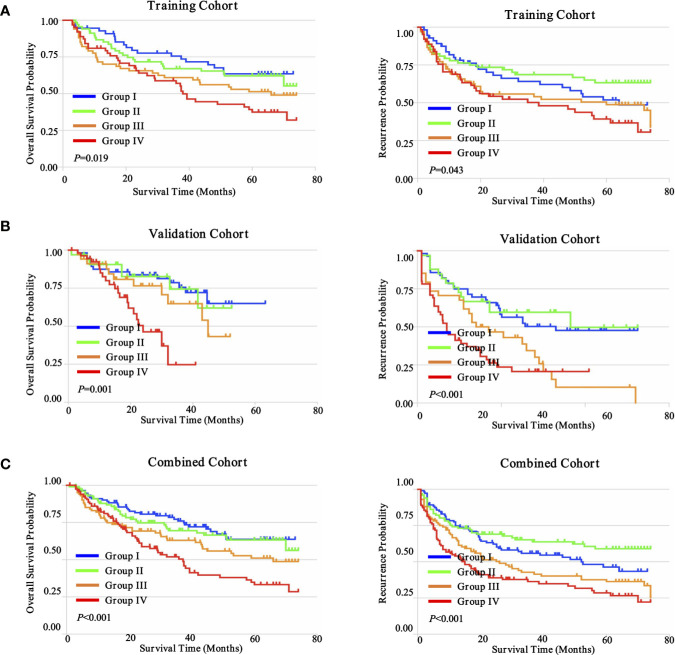
Prognostic significance of distinct immune subtypes in ZS-HCC cohorts. **(A)**. Cumulative OS and TTR were calculated using the Kaplan-Meier method and *P* value was calculated by the log-rank test among sub-types. Patients were divided into four groups according to the median proportion of CD4^+^ T cells, CD8^+^ T cells, and CD56^+^ NK cells in intratumoral tissues in training cohort (n = 258). **(B)**. Validation cohort (n = 178). **(C)**. Combined cohorts (n = 436).

The goal of tumor-associated immunotherapy is to successfully stimulate anti-tumor responses and inhibit tumor-mediated immunosuppression. Anti-tumor immunity strategy has been accomplished through different modalities including cellular immunotherapy, specific vaccines, monoclonal antibodies, and oncolytic virotherapy. The existence and composition of local immune status may affect the activity and migration of tumor associated lymphocytes (TILs) during tumor progression and tumor-mediated immunosuppression (28). To further explore this hypothesis, we further evaluated the potential prognostic implications of combined immune status. As depicted in **Figure 8**, we investigated four distinct immune subtypes of combined immune infiltrates and complex associations with clinical outcomes, according to the previous three immune subgroups, which were significantly correlated with prognosis (CD4^+^ T cells, CD8^+^ T cells, and CD56^+^ NK cells). The immune-hot subtype harbored lower infiltration of CD4^+^ T cells, but higher densities of CD8^+^ T cells and CD56^+^ NK cells, and was further defined as group I. Conversely, immune-cold subtype had a higher infiltration of CD4^+^ T cells, but lower densities of CD8^+^ T cells and CD56^+^ NK cells, and was defined as group IV. The immune-intermediate subtype indicated both higher infiltration of CD4^+^ T cells, CD8^+^ T cells, and CD56^+^ NK cells (group II), or both lower densities CD4^+^ T cells, CD8^+^ T cells, and CD56^+^ NK cells (group III). In the training cohort, patient stratification based on these four groups presented that the immune-hot subtype was associated with improved prognosis, whereas the immune-cold subtype was associated with dismal prognosis, and immune-intermediate subtype was associated with medium prognosis (OS, *P* = 0.019; TTR, *P* = 0.043) (**Figure 8A**). Correlation with clinical outcomes in validation further confirmed that patients with immune-hot subtype had a better survival rate (OS, *P* = 0.001; TTR, *P* < 0.001) (**Figure 8B**). Interestingly, we next interrogated the combined cohort with patient prognosis, and similar prognostic significance of these immune statuses was also confirmed (**Figure 8C**). Taken together, the distinct immune status displayed opposite regulatory directions and this further supported the crucial role of immune imbalance in HCC.

### Inter-Tumoral and Intra-Tumoral Immune Heterogeneity in HCC

In light of the evidence, it can be deduced that the complexity of the tumor, including the intra-tumoral and inter-tumoral heterogeneity, is the major cause of drug resistance and treatment failure (29). To construct a rigorous approach for assessing the spatial immune heterogeneity by histological biomarker expression with accuracy and reproducibility is indeed required.

**Figure 9:**
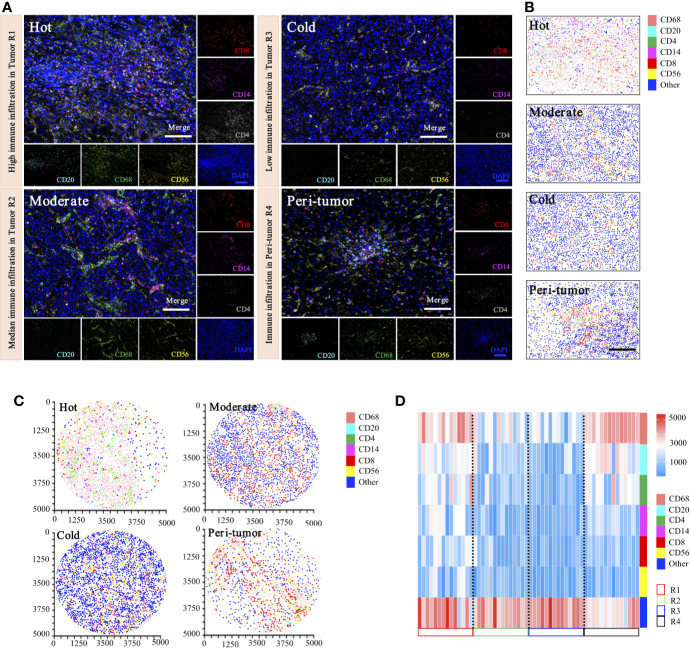
Phenotyping of immune cell density-based subgrouping enables the evaluation of immune heterogeneity in HCC. **(A)**. Representative images to show composite and panel of single spectral image for CD8 (red), CD68 (green), CD4 (white), CD56 (yellow), CD20 (cyan), CD14 (magenta), and DAPI (blue), with three distinct immune infiltration status in intra-tumor centers (R1, Hot; R2, moderate; R3, cold), compared with matched peri-tumor area (R4). Scale bars, 100 μm. **(B)**. Representative phenotype maps indicated three distinct immune infiltration status (Hot, Moderate, and Cold) in intra-tumor tissues and corresponding peri-tumor area, with machine learning algorithm to virtually define *in situ* contexts. Scale bars, 100 μm. **(C)**. Image cytometry-based quantification show multiplex IHC phenotyping findings, indicating high TILs infiltration in R1 (Hot), moderate TILs infiltration in R2 (Moderate), low TILs infiltration in R3 (Cold) and corresponding peri-tumor area (R4). Image plots depict location of cells were identified by image cytometry. CD8^+^ T cell (red), CD68^+^ macrophage (pink), CD4^+^ T cell (green), CD56^+^ NK cell (yellow), CD20^+^ B cell (cyan), CD14^+^ monocyte (magenta), and other cells (blue). **(D)**. Heatmap presents densities of six distinct immune features in 14 HCC patients.

To date, there have been no attempts to systematically map and quantify immune heterogeneity in tandem with coupled protein expression in histological preparations. To determine the inter-tumoral and intra-tumoral immune heterogeneity of HCC in our cohorts, we have optimized and applied a statistical methodology to systematically quantify the spatial immune heterogeneity in whole HCC tissue sections, based on the multiplex imaging system, quantification platform, and unsupervised hierarchical clustering analysis. Three spatial intra-tumoral regions (defined as R1, R2, and R3) and one peri-tumor area (defined as R4) were sampled at multiple sites from 14 HCC cases, including 42 intra-tumoral tissues and 14 peri-tumor tissues, and were selected randomly for heterogeneity exploration (**Figure 9A**).

Representative images of immunohistochemical variables are shown in **Figure 9A**. For intra-tumoral immune heterogeneity, using the peri-tumor area as a reference datum, immune cell infiltration was observed to present a non-homogeneous density in different tumor regions. Three immune subgroups were visualized for exhibiting distinct and characteristic immune cell patterns. The immune-hot presented the most TILs, whereas the immune-cold indicated sparse TILs infiltration, and the immune-moderate meant intermediate infiltration of TILs (**Figure 9B**). Furthermore, image cytometry-based quantification illustrated multiplex phenotyping findings, indicating that high infiltration of TILs was found in R1 (immune-hot), low TILs infiltration in R2 (immune-cold), and median TILs infiltration in R3 (immune-moderate) and the corresponding peri-tumor area (R4) (**Figure 9C**).

For inter-tumoral immune heterogeneity, immune cells infiltrated into HCC tumor centers in a diffuse manner or in lymphoid aggregates, but more abundant cells were in peri-tumoral areas. Significant discrepant infiltrating density of CD4^+^ T cells, CD8^+^ T cells, CD20^+^ B cells, CD14^+^ monocytes, CD56^+^ NK cells, and CD68^+^ macrophages were found within different tumors, excluding necrotic, hemorrhagic, and fibrotic components. Composition analysis indicated the presence of differential immune profiles between tumors and peri-tumor tissues (**Figure 9D**). As highlighted in our immune analysis, HCC had variable TME compositions, with immune signatures impacting observed protein expression patterns.

## Discussion

Precise immunotherapy requires a comprehensive understanding of its micro-environmental complexities and heterogeneities (30). It remains largely undefined how tumor biological behavior is influenced by microenvironment characteristics (31). We revealed the landscape of immune characteristics and complex associations with clinical outcomes using different subgroups and immunomodulators from over 800 HCC cases. We found that more activated CD4^+^ T cells and central memory CD4^+^ T cell subpopulations were associated with a dismal prognosis, but more activated NK and CD56^dim^ NK cells were associated with a favorable prognosis in patients with HCC. When pairs of these three variables (CD4^+^ T cells, CD8^+^ T cells, and CD56^+^ NK cells) were combined and subjected to survival analyses, our analysis showed that immune-hot subtypes harboring lower infiltration of CD4^+^ T cells, but higher densities of CD8^+^ T cells and CD56^+^ NK cells, had the longest survival. These results suggest that systematic analysis of several tumor-infiltrating immune/inflammatory cells is essential for establishing a prognostic model for HCC.

From an immunologic standpoint, the coordination among particular cell types can affect the immune microenvironment. The prognostic values of these selected immune profiles (CD4^+^ T cells, CD8^+^ T cells, and CD56^+^ NK cells) were consistent with previous studies (32). Recently, the prognostic roles of CD4^+^ T cells in tumors have received extensive attention but remain rather controversial. Previous studies indicated a favorable role of CD4^+^ tumor-infiltrating lymphocytes in melanoma (33), colon (34), breast (35), and HCC (36). In contrast, our analysis supports the idea that more infiltration of activated CD4^+^ T cells and central memory CD4^+^ T cells were associated with dismal prognosis in HCC, which is similar to several studies on melanoma (8), colo-rectal (9), and breast cancers (10). This inconsistency may be due to factors such as the difference of methodology, sample size, race, age, gender, and treatment. In line with a previous study (37), a higher density of CD8^+^ T cells was significantly associated with favorable OS in HCC patients. Similar to the previous study (38), more CD56^+^ NK cell infiltration in the intra-tumoral area was significantly associated with better OS and TTR. These findings, together with our present results, suggest that CD4^+^ T cells, CD8^+^ T cells, and CD56^+^ NK cells are the hallmarks of an immune microenvironment.

Monocytes that have diminished or no HLA-DR expression, called CD14^+^HLA-DR^lo/neg^ monocytes, have emerged as important mediators of tumor-induced immunosuppression (39). MDSCs are a heterogeneous population of cells that usually expand on pathological conditions of cancer, inflammation, and infection, and have a remarkable ability to suppress T-cell responses (40). Therefore, MDSCs are commonly referred to as monocytic myeloid derived suppressor cells (41). In contrast to extensive studies on murine MDSCs, human MDSCs are inadequately characterized by few uniform markers, which comprise of two subsets: CD14^+^ monocytic subpopulation (Mo-MDSCs) and CD15^+^ granulocytic subpopulation (G-MDSCs) (42). Although MDSCs were not investigated directly in our study, MDSCs were closely correlated with other subgroups, such as Tregs, monocytes, and macrophages (43). The coordination of MDSCs and the other immune cells still needs further investigation in HCC.

The expression profiles and prognostic significance of other immune phenotypes were also investigated. In HCC, tumor-associated B lymphocytes presented both tumor-promoting and anti-tumor effects (44). In our study, though no significant prognostic difference was observed, the decreased distribution of infiltrating CD20^+^ B cells in intra-tumoral tissues, compared with that in peri-tumoral tissues, was found, suggesting B cells’ potential antitumor effect in the HCC microenvironment (16). Conversely, intra-tumoral neutrophils were defined as a dismal indicator for HCC patients with sorafenib resistance in the previous study (45). However, no significant prognostic values were found in mRNA levels of neutrophils, eosinophils, dendritic cells, macrophages, and monocytes, according to our analysis of transcriptome sequencing results. In the previous studies, macrophages were divided into two phenotypes (46). The classical M1 macrophage was defined with inflammatory response and antitumor immunity, while the alternative M2 macrophage was implicated in anti-inflammatory response and pro-tumorigenic properties in an immune-suppressive microenvironment (47). In our study, neither mRNA levels of M1 nor that of M2 predicted significant prognosis.

Furthermore, we investigated 22 key immune checkpoint modulators in HCC, since these co-stimulatory or inhibitory modulators, especially CTLA-4, PD-1, or PD-L1, may sustain or restrict the anti-tumoral potency of tumor-associated T cells (48). Surprisingly, among 22 modulators, only high levels of OX40 and OX40L were significantly associated with poor prognosis. OX40 is a recently identified T-cell costimulatory molecule belonging to the TNF/TNFR superfamily. It can express in both activated T effector cells and Foxp3^+^ Tregs (49). OX40 can act as a key negative regulator of Foxp3^+^ Tregs and may have important clinical implications in models of transplantation and autoimmunity and in HCC. We proposed that adding immune checkpoint modulators to an immune prognostic model will improve the accuracy of its prognostic power, which needs further investigation.

HCC is defined as a remarkably heterogeneous type of malignancy (50). Consequently, the heterogeneity at epigenetic, genomic, transcriptome, and proteogenomic from the single cell to the lesion hinders the efficacy of treatment, due to the immune escape or drug resistance (29). Consistent with our previous studies, multi-regional sampling and high-throughput analyses in the present study further confirmed intra-tumoral heterogeneity in HCC (13). Analysis of tumor spatial heterogeneity at the single-cell level can reveal distinct features in cancer habitats that indicate different ecological processes. Therefore, comprehensive investigation of the immune heterogeneity of HCC and study of their associations with cancer prognosis are considerately important.

Extensive literature has investigated the host immune response to cancer and demonstrated the prognostic impact of the *in situ* immune cell infiltrating into tumors (51). Immunoscore presented the advanced feasibility and reliable reproducibility in prognostic power, therefore it has been demonstrated to be a prognostic factor superior to the AJCC/UICC TNM classification (52). We used RNA sequence data, multiplex IHC, and immunofluorescence to investigate immune profiles (53). In addition, our study illustrated immune profiles by recruiting two larger cohorts, including 436 HCC patients. Furthermore, an expression landscape of 22 immunomodulators and intra-tumoral heterogeneity were additionally evaluated in our study. Our studies expand the current knowledge on the association between immunological profiles and clinical significance in HCC.

Our study has several limitations. First, we established an immune-related prognostic model of HCC based on transcriptome levels and multiplex immunohistochemistry of six immune subsets. However, immune checkpoint modulators are lacking in our model. Second, although we confirmed the intra-tumoral and inter-tumoral immune heterogeneity in HCC, the correlations between these heterogeneities and prognosis were not analyzed. Third, our study only focuses on the six immune subsets, which cannot represent the landscape of immune cells. Fourth, the CIBERSORT-based analysis of activated/central memory states alone is not enough to make robust conclusions; more validations would be required. In addition, the prognostic value of CD4 in this study is limited because of the lack of Foxp3 co-staining, which needs further investigation.

In summary, this comprehensive study not only shows the immune landscape and heterogeneity in HCC, but also creates new opportunities to carry out an integrated and in-depth study in the near future. Further investigation of specific functions in TILs with distinct immune checkpoint modulators will help us to further understand the immune system in HCC.

## Conclusion

Our findings provide a conceptual framework to understand the immune landscape within HCC. Future work is needed to evaluate its relevance in the design of combination treatment strategies and guiding optimal selection of patients for immunotherapy.

## Data Availability Statement

The datasets presented in this study can be found in online repositories. The names of the repository/repositories and accession number(s) can be found in the article/**Supplementary Material**.

## Ethics Statement

The studies involving human participants were reviewed and approved by Ethics committee board of Zhongshan Hospital, Fudan University (Reference Number: y2017-179). The patients/participants provided their written informed consent to participate in this study.

## Author Contributions

Conception and design: PT, SZ, and LM. Development of methodology: PT, RX, WT, SZ, and LM. Acquisition of data (acquired and managed patients, provided facilities, etc.): PT, LH, RX, SZ, and LM. Analysis and interpretation of data (e.g., statistical analysis, biostatistics, computational analysis): PT, RX, QL, YZ, and LM. Writing, review, and/or revision of the manuscript: PT. Administrative, technical, or material support (i.e., reporting or organizing data, constructing databases): PT, RX, SZ, and LM. Study supervision: RX and LM. All authors contributed to the article and approved the submitted version.

## Funding

This work was supported by the National Natural Science Foundation of China (No.81802302, 81871934, 81770137, and 81702306), Scientific research project of Shanghai TCM-integrated Hospital (No. 18-01-03), and Scientific research project of Hongkou District Health Committee (No. 2003-02).

## Conflict of Interest

The authors declare that the research was conducted in the absence of any commercial or financial relationships that could be construed as a potential conflict of interest.

## Supplementary Material

Supplementary Figure 1 The cellular characteristics associated with the innate immune subsets. **(A)** Comparison of innate immune cells between tumor and normal tissues in TCGA-LIHC cohort. *** denotes *P* < 0.001. NS denotes no significance (Mann-Whitney test).

Supplementary Figure 2 Prognostic landscape of adaptive TME signatures in TCGA-LIHC cohort (n = 370). Kaplan-Meier survival curves of tumor-infiltrating immune subsets in TCGA-LIHC. **(A–M)** Each indicating activated CD8^+^ T cell, central memory CD8^+^ T cell, effector memory CD8^+^ T cell, activated B cell, memory B cell, immature B cell, Effector memory CD4^+^ T cell, regulatory T cell, gamma delta T cell, T follicular helper cell, Type 1 helper cell, Type 2 helper cell and Type 17 helper cell, respectively.

Supplementary Figure 3 Prognostic landscape of innate TME signatures in TCGA-LIHC cohort (n = 370). Kaplan-Meier survival curves of tumor-infiltrating immune subsets in TCGA-LIHC. **(A–I)** Each indicating activated CD56^bright^ NK cell, neutrophil, activated dendritic cell, plasmacytoid dendritic, mast cell, myeloid-derived suppressor cell, monocyte, macrophage, eosinophil, respectively.

Supplementary Figure 4 The cellular characteristics associated with specific ligands of immune checkpoints in TCGA-LIHC cohort (n = 370). **(A)** Comparison of specific ligands of immune checkpoints between tumor and normal tissues in TCGA-LIHC cohort. **, *** denote *P* < 0.01 and *P* < 0.001, respectively. NS denotes no significance (Mann-Whitney test). **(B)** Forest plot showing the results of multivariate Cox regression analysis of 11 selected specific ligands of immune checkpoints in LIHC. * denotes *P* < 0.05.

Supplementary Figure 5 Prognostic landscape of immune regulators in TCGA-LIHC cohort (n = 370). **(A–V)** Kaplan-Meier survival curve of PD-1, CTLA-4, LAG-3, TIM-3, VISTA, CD28, CD40L, OX40, 4-1BB, ICOS, GITR, PD-L1, PD-L2, CD80, CD86, HLA-DRB1, Galectin-9, CD40, OX40L, 4-1BBL, ICOSL and GITRL. *P* value was calculated by the log-rank test.

Supplementary Figure 6 Visualizing the correlation of expression of immune checkpoints with immune infiltration level in TCGA-LIHC cohort (n = 370). The scatter-plots was generated and displayed as showing the purity-corrected partial Spearman’s correlation and statistical significance. The gene expression levels against tumor purity are always displayed on the left-most panel. Genes highly expressed in the microenvironment have negative associations with tumor purity. **(A**, **B)** Immune checkpoints.

Supplementary Figure 7 Kaplan–Meier curves for OS and TTR of all patients stratified by the immune subtypes in ZS-HCC training cohort (n = 258). **(A–C)** CD20, CD68 and CD14, respectively.

Supplementary Figure 8 Kaplan–Meier curves for OS and TTR of all patients stratified by the immune subtypes in ZS-HCC validation cohort (n = 178). **(A–F)** CD4, CD20, CD68, CD14, CD8 and CD56, respectively.
