# Supplementary figures and images for: Comprehensive Characterization of Immunological Profiles and Clinical Significance in Hepatocellular Carcinoma
Source: Front Oncol. 2021 Jan 22;10:574778. doi: 10.3389/fonc.2020.574778 (PMC7862794; doi:10.3389/fonc.2020.574778)

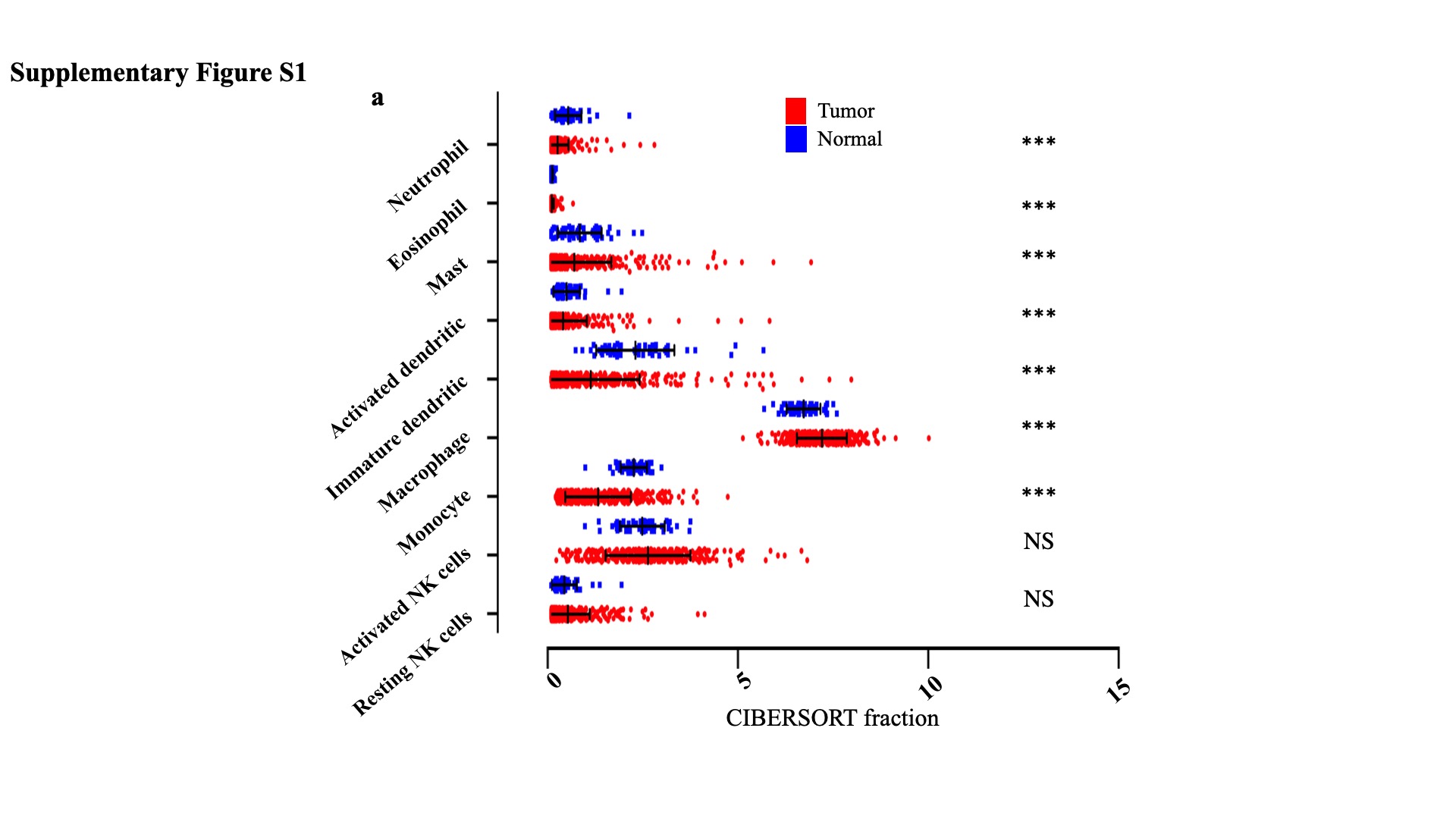

Supplement: Supplementary Figure 1 — The cellular characteristics associated with the innate immune subsets. (A) Comparison of innate immune cells between tumor and normal tissues in TCGA-LIHC cohort. *** denotes P < 0.001. NS denotes no significance (Mann-Whitney test). [file Image_1.jpeg]

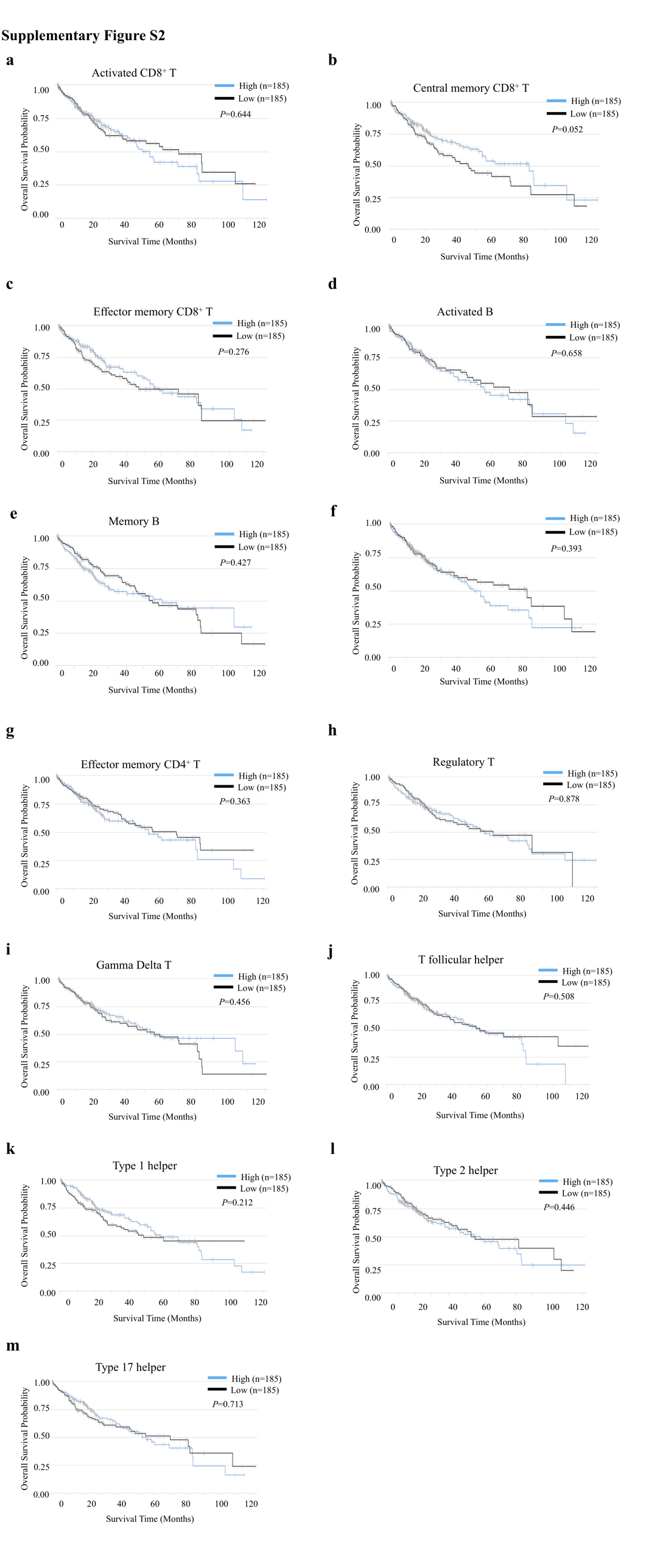

Supplement: Supplementary Figure 2 — Prognostic landscape of adaptive TME signatures in TCGA-LIHC cohort (n = 370). Kaplan-Meier survival curves of tumor-infiltrating immune subsets in TCGA-LIHC. (A–M) Each indicating activated CD8+ T cell, central memory CD8+ T cell, effector memory CD8+ T cell, activated B cell, memory B cell, immature B cell, Effector memory CD4+ T cell, regulatory T cell, gamma delta T cell, T follicular helper cell, Type 1 helper cell, Type 2 helper cell and Type 17 helper cell, respectively. [file Image_2.jpeg]

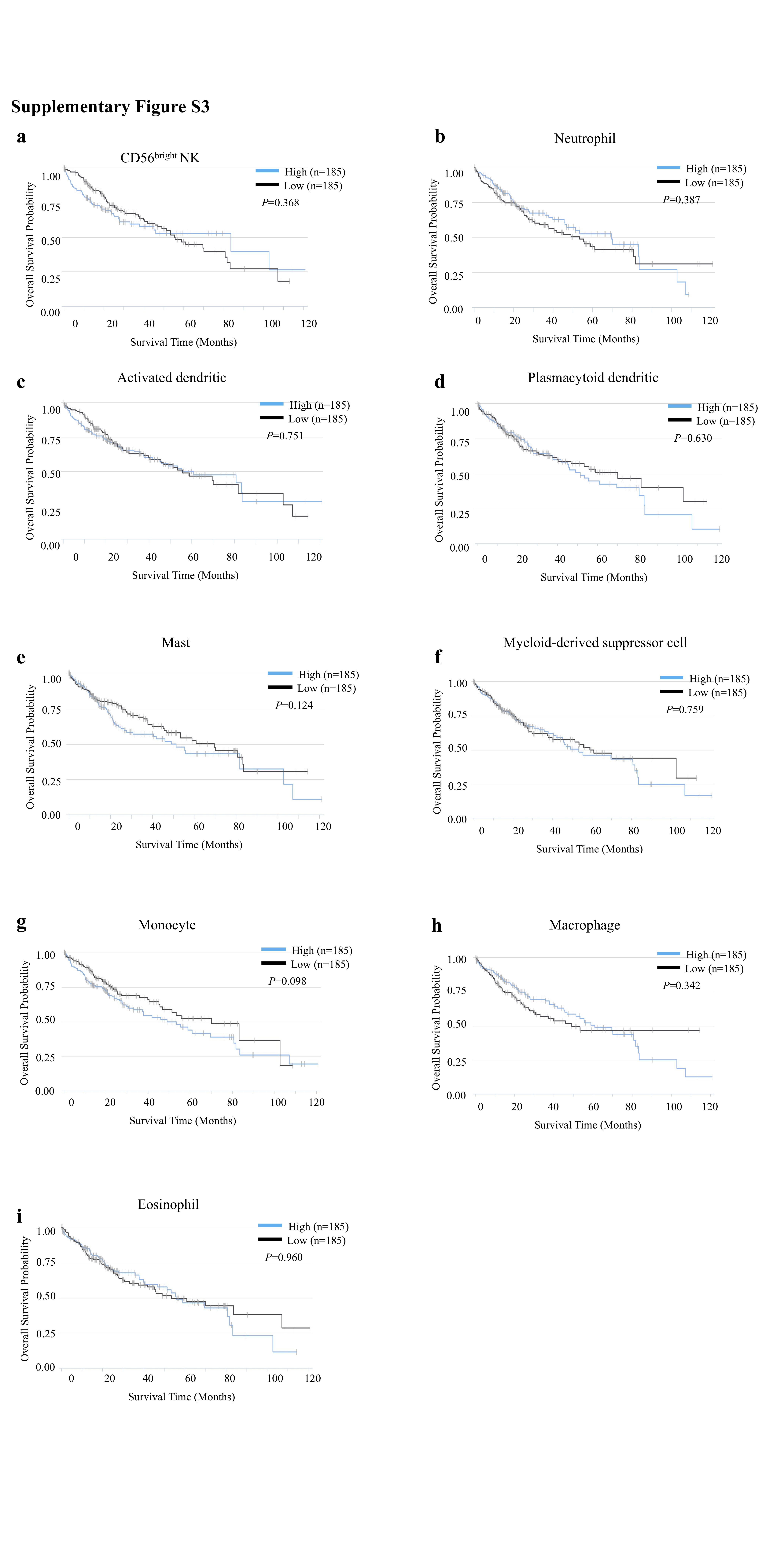

Supplement: Supplementary Figure 3 — Prognostic landscape of innate TME signatures in TCGA-LIHC cohort (n = 370). Kaplan-Meier survival curves of tumor-infiltrating immune subsets in TCGA-LIHC. (A–I) Each indicating activated CD56bright NK cell, neutrophil, activated dendritic cell, plasmacytoid dendritic, mast cell, myeloid-derived suppressor cell, monocyte, macrophage, eosinophil, respectively. [file Image_3.jpeg]

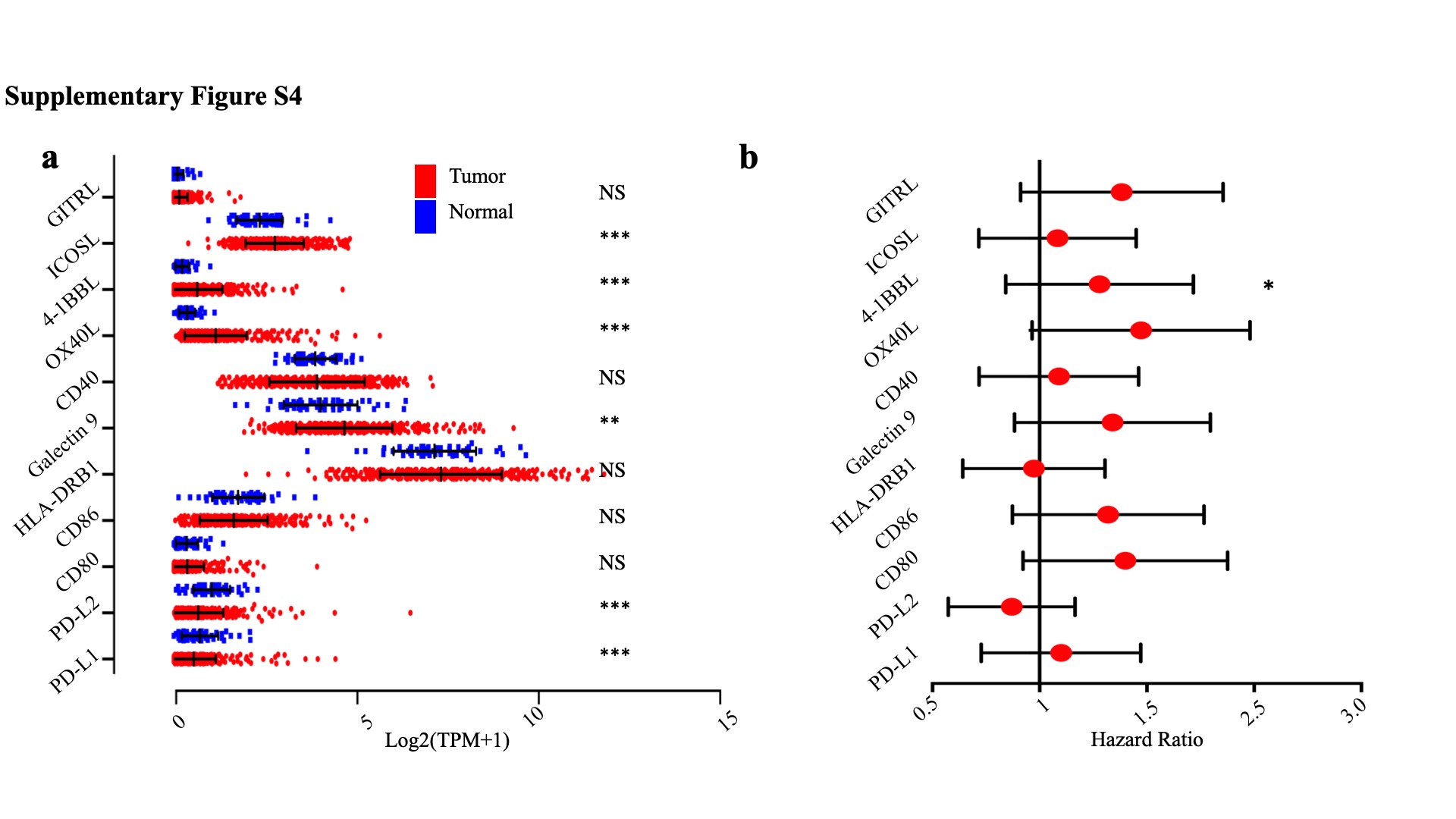

Supplement: Supplementary Figure 4 — The cellular characteristics associated with specific ligands of immune checkpoints in TCGA-LIHC cohort (n = 370). (A) Comparison of specific ligands of immune checkpoints between tumor and normal tissues in TCGA-LIHC cohort. **, *** denote P < 0.01 and P < 0.001, respectively. NS denotes no significance (Mann-Whitney test). (B) Forest plot showing the results of multivariate Cox regression analysis of 11 selected specific ligands of immune checkpoints in LIHC. * denotes P < 0.05. [file Image_4.jpeg]

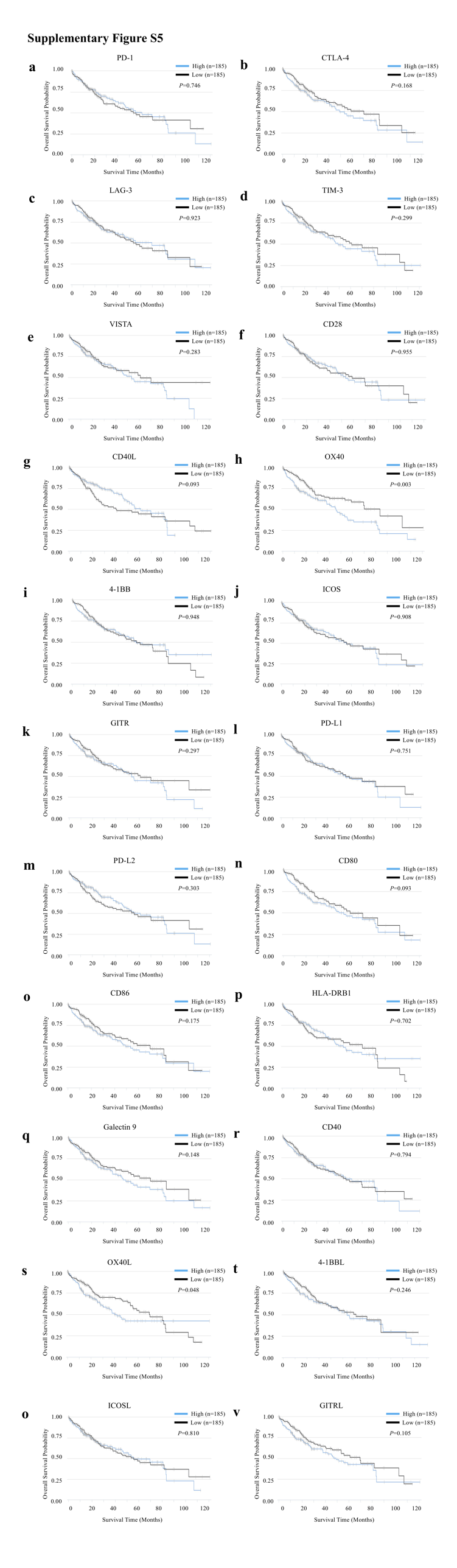

Supplement: Supplementary Figure 5 — Prognostic landscape of immune regulators in TCGA-LIHC cohort (n = 370). (A–V) Kaplan-Meier survival curve of PD-1, CTLA-4, LAG-3, TIM-3, VISTA, CD28, CD40L, OX40, 4-1BB, ICOS, GITR, PD-L1, PD-L2, CD80, CD86, HLA-DRB1, Galectin-9, CD40, OX40L, 4-1BBL, ICOSL and GITRL. P value was calculated by the log-rank test. [file Image_5.jpeg]

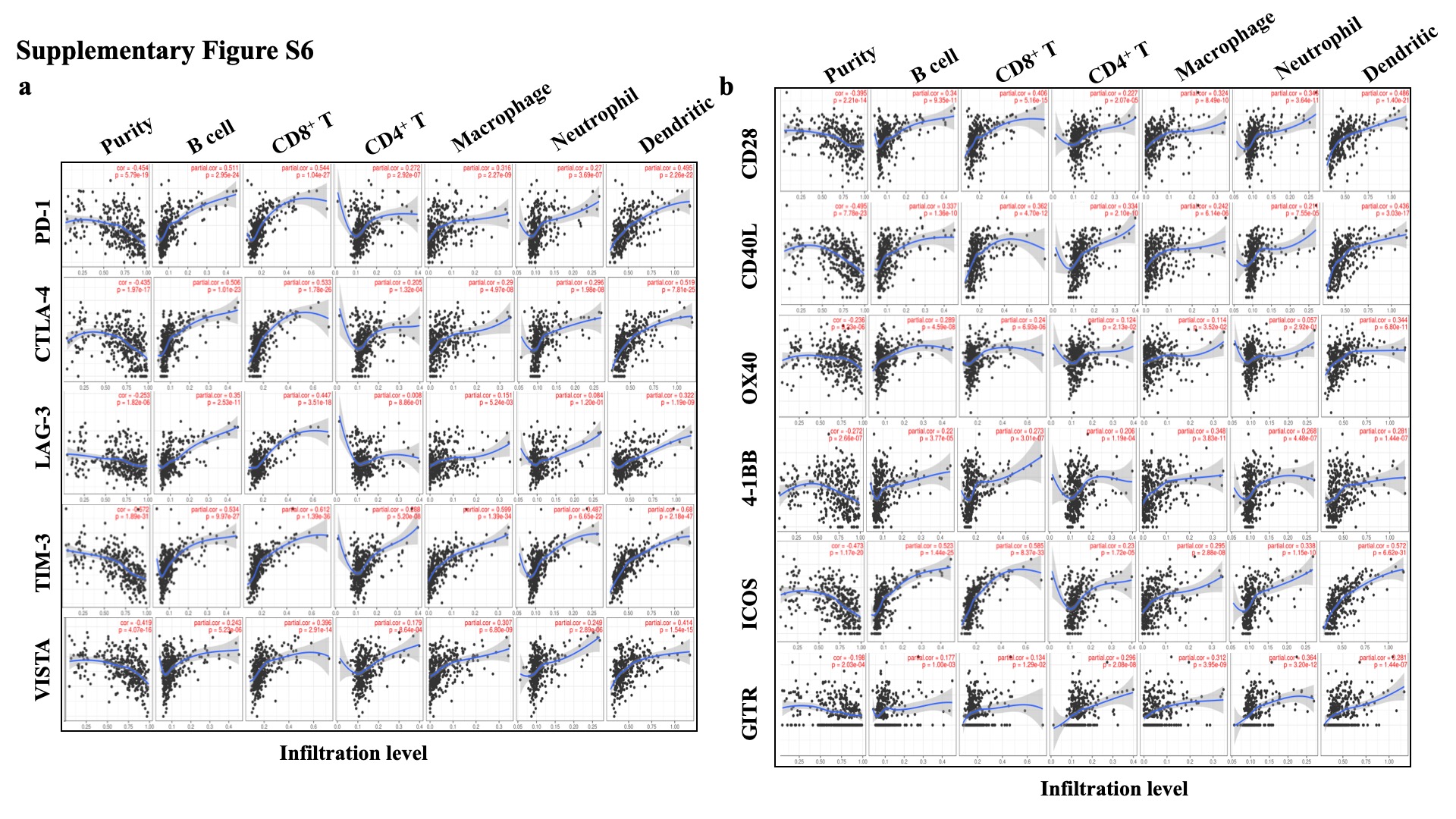

Supplement: Supplementary Figure 6 — Visualizing the correlation of expression of immune checkpoints with immune infiltration level in TCGA-LIHC cohort (n = 370). The scatter-plots was generated and displayed as showing the purity-corrected partial Spearman’s correlation and statistical significance. The gene expression levels against tumor purity are always displayed on the left-most panel. Genes highly expressed in the microenvironment have negative associations with tumor purity. (A, B) Immune checkpoints. [file Image_6.jpeg]

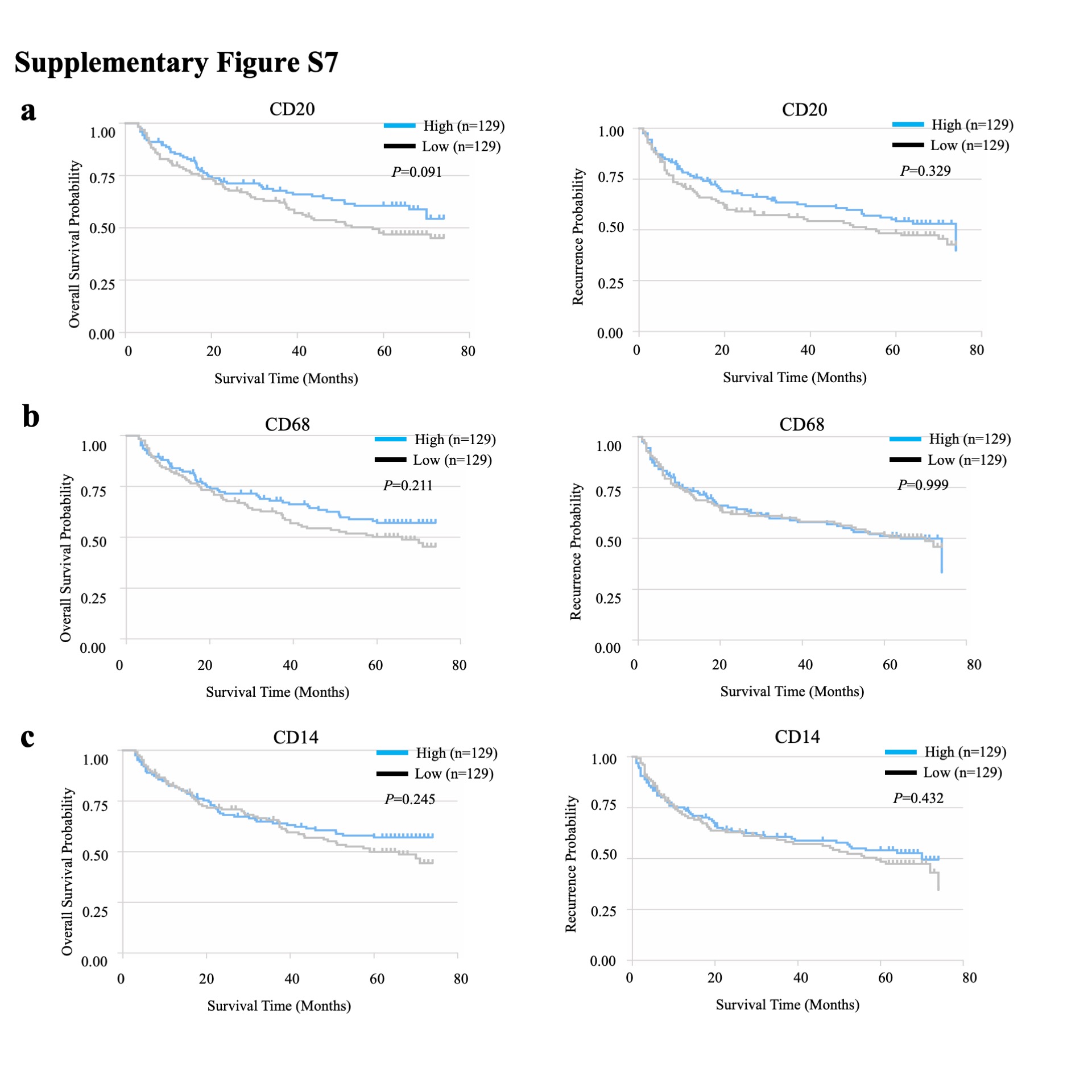

Supplement: Supplementary Figure 7 — Kaplan–Meier curves for OS and TTR of all patients stratified by the immune subtypes in ZS-HCC training cohort (n = 258). (A–C) CD20, CD68 and CD14, respectively. [file Image_7.jpeg]

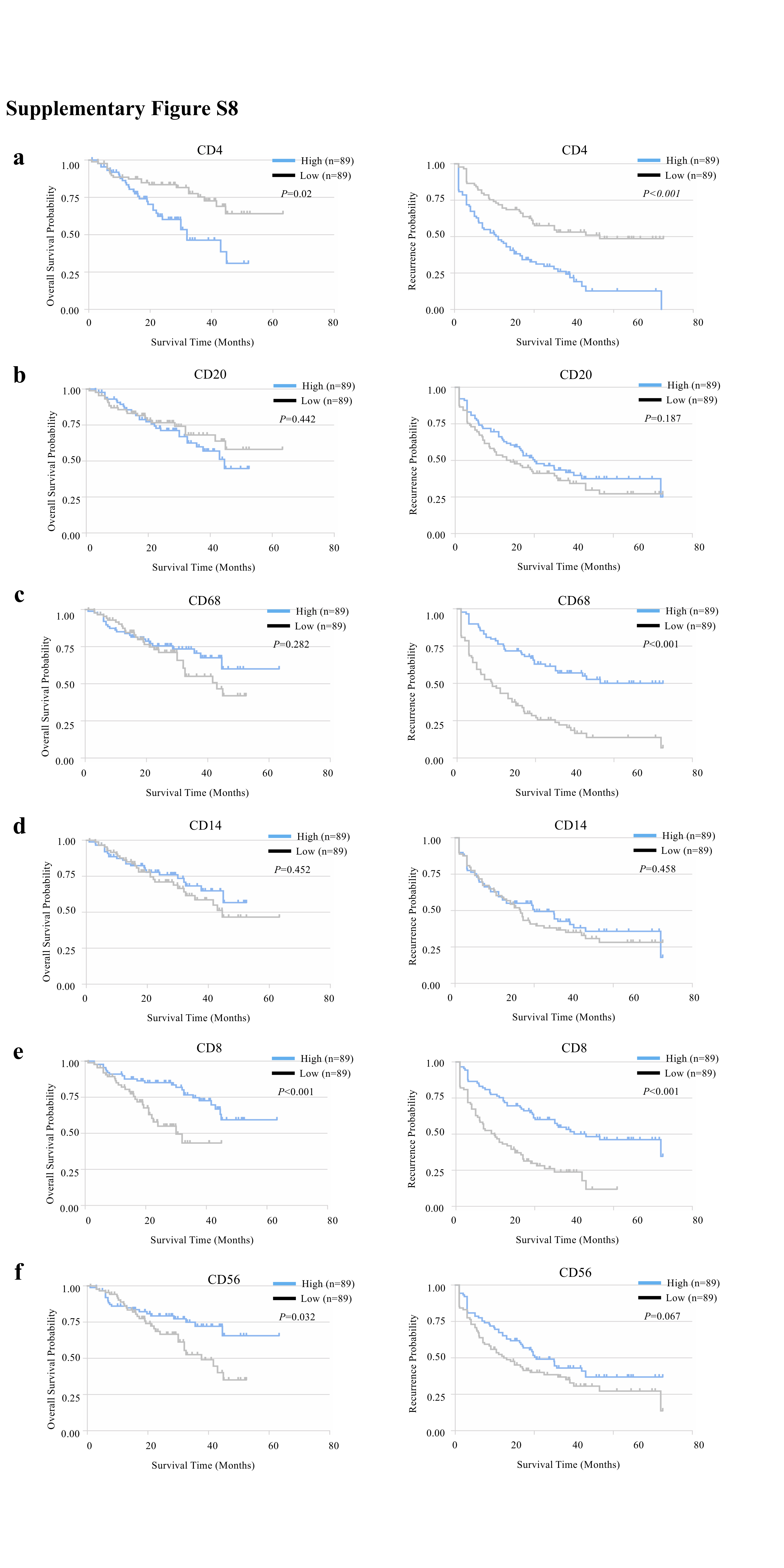

Supplement: Supplementary Figure 8 — Kaplan–Meier curves for OS and TTR of all patients stratified by the immune subtypes in ZS-HCC validation cohort (n = 178). (A–F) CD4, CD20, CD68, CD14, CD8 and CD56, respectively. [file Image_8.jpeg]
