# Supplementary material for: Comprehensive Characterization of Immunological Profiles and Clinical Significance in Hepatocellular Carcinoma
Source: Front Oncol. 2021 Jan 22;10:574778. doi: 10.3389/fonc.2020.574778 (PMC7862794; doi:10.3389/fonc.2020.574778)
Supplement: Supplementary file 9 [file Table_1.docx]

**Supplementary Table S1. The clinicopathological characteristics of Zhongshan cohorts and TCGA-LIHC cohort.**

| **Variables** | **Training cohort**  **(n=258)** | **Validation cohort (n=178)** | **TCGA-LIHC**  **cohort (n=370)** |
| --- | --- | --- | --- |
| Gender  (Male vs. Female) | 220/38 | 155/23 | 253/117 |
| HbsAg  (Negative vs. Positive) | 9/249 | NA | NA |
| Serum ALT, U/L  (>75 vs. ≤75) | 28/230 | 66/112 | NA |
| Serum AFP (ng/ml)  (>20 vs. ≤20) | 154/104 | 116/62 | NA |
| Tumor size (cm)  (≤5 vs. >5) | 114/144 | 73/105 | NA |
| Tumor multiplicity  (Multiple vs. Single) | 45/213 | 27/151 | NA |
| Tumor differentiation  (Poor vs. Well) | 181/77 | 108/70 | NA |
| Microvascular invasion  (Yes vs. No) | 90/168 | 63/115 | NA |
| TNM stage  (II-III vs. I vs. NA) | 115/143 | 68/110 | 174/174/22 |
| Death  (Yes vs. No) | 116/142 | 53/125 | 127/243 |

***Abbreviation:*** AFP, alpha-fetoprotein; ALT, alanine aminotransferase; TNM, tumor-nodes-metastases.
