# Supplementary material for: Comprehensive Characterization of Immunological Profiles and Clinical Significance in Hepatocellular Carcinoma
Source: Front Oncol. 2021 Jan 22;10:574778. doi: 10.3389/fonc.2020.574778 (PMC7862794; doi:10.3389/fonc.2020.574778)
Supplement: Supplementary file 11 [file Table_3.docx]

**Supplementary Table S3.** **Sequential opal multiplex staining protocol of IHC.**

| Antigen | Antigen retrieval | | Primary antibody | | Catalogue number | Secondary polymer | | TSA fluorophore |
| --- | --- | --- | --- | --- | --- | --- | --- | --- |
|  |  |  | Concentration | Supplier |  | Polymer | Supplier |  |
| CD20 | Citrate | 10mins | 1:2000 | eBioscience | 14-0202-82 | Anti-MOUSE IgG | VECTOR | Opal 520 |
| CD56 | Citrate | 10mins | 1:2000 | Abcam | Ab75813 | Anti-RABBIT IgG | VECTOR | Opal 540 |
| CD4 | Citrate | 10mins | 1:2000 | Abcam | Ab183685 | Anti-RABBIT IgG | VECTOR | Opal 570 |
| CD68 | Citrate | 10mins | 1:2000 | Abcam | Ab955 | Anti-MOUSE IgG | VECTOR | Opal 620 |
| CD14 | Citrate | 10mins | 1:2000 | Abcam | Ab183322 | Anti-RABBIT IgG | VECTOR | Opal 650 |
| CD8 | Citrate | 10mins | 1:2000 | Dako Omnis | IR623 | Anti-MOUSE IgG | VECTOR | Opal 690 |
